# P-1603. COVID-19 Case Attack Rate Differences by Vaccination Status and Vaccine Effectiveness among US Nursing Home Residents, October 5, 2024 to January 5, 2025

**DOI:** 10.1093/ofid/ofaf695.1782

**Published:** 2026-01-11

**Authors:** Farid L Khan, Evan Zasowski, Angela Cook, Jenny Boucher, Tobias Bergroth, Santiago M C Lopez, Timothy L Wiemken, Laura A Puzniak

**Affiliations:** Pfizer, Collegeville, PA; Pfizer, Collegeville, PA; Pfizer, Collegeville, PA; Pfizer, Collegeville, PA; Pfizer, Collegeville, PA; Pfizer Inc, Collegeville, Pennsylvania; Pfizer Inc, Collegeville, Pennsylvania; Pfizer Inc., Collegeville, Pennsylvania

## Abstract

**Background:**

US nursing home residents have experienced a disproportionate burden of COVID-19-associated cases. Vaccination against SARS-CoV-2 had previously demonstrated a reduction in burden, however, recent data on vaccine effectiveness in this vulnerable population are lacking.

**Methods:**

A study was conducted using data from the CDC’s National Healthcare Safety Network (NHSN) Long-Term Care Facility (LTCF) COVID-19 Module database, covering October 5, 2024, to January 5, 2025. Facility-level data on COVID-19 cases among residents who were, or were not, up-to-date with COVID-19 vaccine recommendations (receipt of 2024-2025 COVID-19 vaccine or 2023-2024 COVID-19 vaccine within the prior 2 months) were utilized to compare attack rates (COVID-19 cases per 100,000 resident population by respective up-to-date status (VAR = vaccinated attack rate; NVAR = unvaccinated attack rate) and calculate unadjusted vaccine effectiveness ((NVAR - VAR) ÷ NVAR).

**Results:**

The study included 12,917 facilities representing1,137,412 residents, of whom 480,934 (42%) were vaccinated as of January 5, 2025. Of 71,780 COVID-19 cases, 58,622 (82%) were not up-to-date with recommended COVID-19 vaccination, while 13,158 (18%) were up-to-date. The attack rates were 8,930 COVID-19 cases per 100,000 not up-to-date residents compared to 2,736 COVID-19 cases per 100,000 up-to-date residents. The estimated unadjusted vaccine effectiveness was 69.4% (95% CI: 68.8% – 69.9%).

**Conclusion:**

COVID-19 vaccination during the first half of the 2024-2025 respiratory season was found to be 69% effective at reducing COVID-19 cases among nursing home residents, reinforcing the importance of keeping current with recommended vaccinations as a public health strategy in this vulnerable population. Aligning Quality Measure ratings for COVID vaccination uptake similar to flu vaccine, may significantly reduce the morbidity and mortality for these high-risk residents of nursing homes.

**Disclosures:**

Farid L. Khan, MPH, Pfizer: Employee|Pfizer: Stocks/Bonds (Public Company) Evan Zasowski, PharmD, Pfizer: Employee|Pfizer: Stocks/Bonds (Public Company) Angela Cook, MS, Pfizer: Employee|Pfizer: Stocks/Bonds (Public Company) Jenny Boucher, PharmD, Pfizer: Employee|Pfizer: Stocks/Bonds (Public Company) Tobias Bergroth, PhD, Pfizer: Employee|Pfizer: Stocks/Bonds (Public Company) Santiago M.C. Lopez, MD, Pfizer Inc.: Employee of Pfizer Inc. and may hold stock or stock options|Pfizer Inc.: Stocks/Bonds (Public Company) Timothy L. Wiemken, PhD, Pfizer: Employee|Pfizer: Stocks/Bonds (Public Company) Laura A. Puzniak, PhD. MPH, Pfizer Inc.: Employee of Pfizer Inc. and may hold stock or stock options|Pfizer Inc.: Stocks/Bonds (Public Company)

